# Solving humanity's grand challenges: Water, food, and energy

**DOI:** 10.3389/frma.2022.1005119

**Published:** 2022-12-02

**Authors:** Vivek Wadhwa

**Affiliations:** Harvard Law School, Cambridge, MA, United States

**Keywords:** abundance, water, food, energy, exponential technologies, technology

Many people are fearful that the future will be one of shortages and scarcity and that because of a burgeoning population and dwindling resources, our future is grim (Wadhwa and Salkever, 2019).

This couldn't be further from reality. This is the most innovative period in human history. With several technologies on exponential and converging paths, we will be able to solve some of humanity's grand challenges—and create an era of abundance. Imagine a world with unlimited food, water, and energy—in which we prevent disease rather than cure it and in which our lifespans increase along with our wisdom and knowledge. This is what is possible, not in future centuries, but in the next two decades.

This may seem like wishful thinking, but consider how far we've come. The majority of people in Asia and Africa now have electrical power, refrigeration, and television. Even the poor have mobile phones. Two hundred years ago, kings and queens didn't have these luxuries. Yes, there is still dire poverty, but there is also hope.

In some fields, such as energy, it is the global momentum that will transform humanity. In others, it will be our scientists and entrepreneurs, and not just those in rich nations. After all, they are the ones who understand the pain and suffering of the world's masses better than the elitist techies in Silicon Valley and the academic researchers in university labs.

The fact is that with the exponential advances in fields like robotics, A.I., synthetic biology, 3D printing, medicine, and nanomaterials, the costs are also dropping, enabling small teams to do what once was possible only for governments and large corporations.

When Google was founded in 1998, for example, the DEC AlphaServer 8400, a minicomputer with the same processing power as first generation iPads, cost close to $1 million. Storage necessitated installing a server farm and rack upon rack of hard disks. It cost millions of dollars to start a technology-based company. Today, anyone can buy computing power and storage for practically nothing from companies such as Amazon, Microsoft, and Google. One iPhone 13 has more computational power than the combined power of all of the Cray supercomputers of yesteryear—which the U.S. placed tight export restrictions on. Today even the poor in the developing world carry supercomputers in their pockets and use them to check WhatsApp messages and make phone calls every now and then.

It cost about three billion dollars to sequence a full human genome in the year 2000. It costs less than five hundred dollars to do now. Soon it will cost less than a cup of coffee. Genome data are available from hundreds of millions of people already; soon this will be in the billions. Anyone anywhere can now write computer code that compares one person's DNA with another; learn what diseases people with similar genes have had; and analyze the correspondences between genomes and the effectiveness with which different medications or other interventions have treated a given disease.

The same advances are happening with sensor-based devices. Sensors such as those in our iPhones cost tens of thousands of dollars a few years ago but now cost practically nothing. The world's entrepreneurs are building iPhone apps that act like medical assistants and detect disease; body sensors that monitor heart, brain, and body activity; external sensors monitor soil humidity, pressure in oil pipelines, and traffic patterns.

So, anyone anywhere can build solutions to humanity's grand challenges—in areas such as health, energy, food, education, water and security—and they are beginning to do just that.

## Water

Take the water crisis. Waterborne viruses are responsible for the majority of disease in the developing world. There are predictions that many countries will run out of water and that wars will break out over supplies. This seems paradoxical considering that 71% of the earth's surface is water and converting seawater is as simple as boiling and condensing vapor.

Access to clean water is one of the most serious problems in the developing world. According to the World Health Organization, 1.8 million people die every year from diarrheal diseases (World Health Organization, 2004). Of these victims, 90% are children under five, mostly in developing countries. Eighty-eight percent of these cases are attributed to unsafe water supply and sanitation.

It's not shortage of water per se that is the problem; it's access to clean water. Water obtained from rivers and wells is infested with deadly bacteria, viruses, and larger parasites. These could be killed by simply boiling the water, but the energy necessary to do that is prohibitively expensive, so people die or suffer.

One incredible Chilean entrepreneur, someone I consider to have the genius of Albert Einstein and inventive capabilities of Thomas Edison, took it upon himself to solve this grand challenge.

Alfredo Zolezzi, of Advanced Innovation Center in Chile, had spent the early part of his career creating products for the oil industry. He had achieved great success as an entrepreneur by developing technology that enhanced the recovery of oil from abandoned oil wells using high-frequency, high-powered ultrasound waves. He had ideas for new technologies that could reduce the cost of refining heavy oil as well as its viscosity and sulfur content. Zolezzi likely could have made billions by perfecting these.

But then, in 2009, he read that the United Nations was discussing a resolution to make access to clean drinking water a basic human right—just like the right to food and freedom. When Zolezzi started researching the issue, he learned waterborne viruses are the leading cause of disease and death around the world—taking an annual toll of more than 3.4 million lives. And he was even more shocked to learn that the suffering weren't just in sub-Saharan Africa. A slum that he visited near his home in Santiago, Chile, had the same problem. Its inhabitants fell sick frequently and spent a substantial part of their earnings on emergency hospital care.

He realized he had lived a privileged life. He came from a middle-class family, had had a good education, and was able to achieve great personal success by using technology to solve the problems of big corporations. Zolezzi says that he realized that he needed to use these gifts to do something for those who have nothing.

So he decided to shift gear and develop a technology to help solve the problem of water purification. He started repurposing his oil-extraction technology to eliminate microbial contaminants from water.

Zolezzi told me he was driven by a social need. But he also believed that he could build a profitable company and achieve entrepreneurial success.

Zolezzi and his team spent 18 months developing a system that converts water into a plasma state through a high-intensity electrical field and eliminates microbiological content through electroporation, oxidation, ionization, UV and IR radiation and shockwaves.

They installed it in the Santiago slum in mid-2011 and I heard about Zolezzi's project when I visited Chile in April 2012 as an advisor on innovation to the Chilean government. When I visited, Rosa Reyes, community leader of the Fundo San Jose shantytown, told me how grateful she was to Zolezzi and his team for transforming their lives. Their productivity had increased. Her neighbors no longer had to keep borrowing money from each other to pay for medical care. Reyes said that the local hospital, fearing that it was losing business to a competitor, had sent a representative to ask why they had stopped frequenting their facility.

I invested in Zolezzi's company and had philanthropists such as Ratan Tata of India and Ricardo Salinas of Mexico do the same.

Zolezzi brought his technology to the U.S. to have it tested for conformance to EPA guidelines by the leading U.S. authority, NSF International. It not only exceeded NSF's highest standards, but killed 100% of all bacteria and viruses in the heavily tainted samples that NSF tested—something they had never seen before.

Village-sized units of the plasma-based water-sanitization (PWSS) technology—that consume less energy than a hairdryer—presently cost about $10,000 to produce but should cost around $1,000 when mass produced.

The company has been disadvantaged because it was based in Chile and few people believed that it the country could develop breakthrough technologies, so they provided little support. Yet, Zolezzi persevered and is about to take these technologies to the world with the products being manufactured by Siemens AG, a German multinational conglomerate.

A technology developed by a small team in Chile could go a long way toward solving one of humanity's greatest problems.

Not surprisingly, it isn't just the poor who stand to benefit from Zolezzi's technology. A study of aircraft water quality, published in the *International Journal of Environmental Research and Public Health* (Handschuh et al., 2015), found that the water tanks are conducive to microbial growth and that the problem is severe on long-haul flights. In 2014, the Environmental Protection Agency also sampled water from planes and found that 15% of the samples contained coliform bacteria, an indicator of poor hygiene (Handschuh et al., 2015). Our hospitals, schools, and businesses often have water tanks with similar problems. Such contamination partly explains why in 2018 consumers worldwide spent more than $250 billion on bottled water (The Business Research Company, 2018).

European aerospace giant Airbus funded the development of a version of the PWSS product that is the size of a suitcase and can work on board planes. These produced the same results as with the PWSS camp units: 100% elimination of bacteria and viruses. We will hopefully see these units in our homes and reduce the demand for bottled water in plastic containers that pollute the environment.

## Agriculture

As I mentioned earlier, things are never easy for entrepreneurs, especially in the less developed parts of the world. Not only are they restrained by the lack of investment capital, they don't get the support they need from their own communities and governments.

This is the problem that Alfredo Zolezzi faced during the COVID pandemic. As always happens with technology commercialization, it took 5 years longer than expected to perfect these and make them scalable. And just as Siemens and Airbus had finalized the development of a small-sized plasma-water unit for aircraft, schools, and hospitals, the pandemic stopped everything. The company out of funds and there was no further runway.

Fortunately, Zolezzi used the pandemic downtime to go back to basics and look for new uses of his technology. He made an astonishing discovery: the devices could inexpensively produce Plasma Activated Water (PAW) in a rapid and continuous flow, just as it produces clean water. PAW may well be the Holy Grail of agriculture because it seems to work like magic by synthesizing compounds that act on plants in the same way as organic pesticides; enhance plant growth; and provide microbial disinfection.

## PAW in agriculture

All of modern agriculture depends upon chemical inputs to control disease and increase yield. Together, these inputs more than quadruple the productivity of much of agriculture, and make it able to support the 8 billion people living today. The two most intensive inputs are the pesticides/microbicides and fertilizers.

Pesticides and fertilizers are also the most toxic and the most polluting. There are more than 1,500 pesticides in use today. All of them require complex synthesis through many chemical intermediates, and all of them generate waste streams that enter the environment. The pesticides themselves are toxic. They are designed to poison and kill bacteria, fungi and insects.

Fertilizer is usually in the form of nitrate. This is produced in massive factories around the world using high pressure, high temperature, and metal catalysts in a process called Haber-Bosch. This on its own produces more than 10% of all atmospheric pollution. The nitrate itself is added to farmers' fields in massive excess, and run-off pollutes rivers and oceans and causes so-called “dead zones” to form around the world.

PAW offers the opportunity to replace at least some of these pesticides and fertilizers with natural and fully-sustainable alternatives. Zolezzi's technology generates hydrogen peroxide from water, and nitrate from air.

Plants including crop plants use hydrogen peroxide to defend themselves from disease. When attacked by a potential pathogen, plant cells make and use peroxide locally to kill the pathogen, and systemically as a signal to build an immune state; hydrogen peroxide triggers the appearance of immunity. PAW, if applied to roots or leaves, should be able to replace at least some toxic microbicides with a fully-sustainable and completely harmless inducer of a natural immune state in crops plants. Then crops wouldn't need toxic sprays because they would be naturally resistant to disease.

Plants including crop plants use nitrate to make amino acids and proteins, and therefore to grow and yield fruits and seeds. The PAW can be made to contain nitrate converted from di-nitrogen in the air. In this way it should be able to replace at least some of the fertilizer made by the Haber-Bosch process with a fully-sustainable and non-harmful source of nitrate. Because it can be applied throughout the season, it can also avoid the massive over-dressing of crop fields that leads to runoff and dead-zones in water courses.

This technology has the potential to displace and disrupt existing pesticide and fertilizer industries worldwide. With scaling, and with commercially-centered trialing and confirmation, there is the potential to use natural and sustainable hydrogen peroxide instead of synthetic pesticide, and natural and sustainable nitrate instead of synthetic fertilizer. If we can do this, we will have a very large impact on the health of the planet and of everyone that lives on it.

## Energy

What blocked our ability to tap the sun until recently was the cost of capturing its energy and converting it into electricity (and, ultimately, heat). But a few things have changed since the 1980s. We have become much better at making semiconductors for computers; and those same pieces of silicon are what convert solar energy into electricity. We have developed ways to make solar panels from thinner slivers of silicon. We have gotten much better also at figuring out how to squeeze more out of the solar energy we capture. And, most important, economies of scale are beginning to affect the price. As more solar panels are installed, more are manufactured, and panel- and -component-manufacture costs keep falling.

For these reasons, solar-energy capture is advancing on an exponential curve. With that advance, we are heading into an era of practically unlimited, clean, almost free energy.

Consider that when Ronald Reagan took office in 1980, average retail electricity costs in the United States were around 5 cents a kilowatt hour (in today's dollars). Electricity produced from wind power, on the other hand, cost around ten times more, at 50 cents a kilowatt hour. And electricity from solar power cost 30 times more, at around $1.50 per kilowatt hour.

How the times have changed. Today, new wind power installations are producing electricity at an unsubsidized cost of 2.6–5.4 cents per kilowatt hour—significantly lower than the 7 cents per kilowatt hour wholesale prices of new coal and natural gas electricity. Solar has dropped as much and is still dropping. Large-scale solar installations in the very sunniest areas range from 2.9 to 3.8 cents per kilowatt hour without subsidies. In fact, at times renewable power is so efficient that utilities literally give it away in order to avoid overloading the grid! In March 2017, over the course of 14 days California utilities gave free power to Arizona utilities to keep the power supply on the California electrical grid in balance (Penn, 2017). This happens in many places where daytime solar or nighttime wind production are so great that utilities have more power than customers can consume.

The first solar photovoltaic panel built by Bell Labs in 1954 cost $1,000 per watt of power it could produce (Chapin et al., 1954). In 2008, modules used in solar arrays cost $3.49 per watt; by 2018, they cost 40 cents per watt (U.S. Energy Information Administration, 2018). According to a pattern known as Swanson's Law, the price of solar photovoltaic modules tends to fall by 20% for every doubling of cumulative shipped volume. The full price of solar electricity (including land, labor to deploy the solar panels, and other equipment required) falls by about 15% with every doubling. In actuality, even this trend is accelerating: Bloomberg New Energy Finance estimates that for every doubling of cumulative manufactured capacity, the cost of PV modules now declines by 28%.

The amount of solar-generated power has been doubling every 2 years or less for the past 40 years—as costs have been falling (Randall, 2016). At this rate, solar power is only five doublings—or <12 years—away from being able to meet 100% of today's energy needs. Power usage will keep increasing, so this is a moving target. Taking that into account, inexpensive renewable sources can potentially provide more power than the world needs in <20 years. This is happening because of the momentum that solar has already gained and the constant refinements to the underlying technologies, which are advancing on exponential curves. What futurist Ray Kurzweil said about Craig Venter's progress when he had just sequenced 1% of the human genome—that Venter was actually halfway to 100% because on an exponential curve, the time required to get from 0.01 to 1% is equal to the time required to get from 1 to 100%—applies to solar capture too.

It isn't just solar production that is advancing at a rapid rate, and solar will not be our only source of clean energy: there are also technologies to harness wind, biomass, thermal, tidal, and waste-breakdown energy, and research projects all over the world are working on improving their efficiency and effectiveness. Wind energy's price became competitive with the cost of energy from new coal-burning power plants in the United States in 2016, according to Bloomberg New Energy Finance, and prices have been continuing to fall (Henbest et al., 2016). Unsubsidized wind-energy contracts were signed at 2 cents per kWh in Mexico and Brazil in late 2017 and early 2018 (Vanessa, 2018).

Yes, there are challenges in the economics and recyclability of solar panels—but these will be solved as these technologies evolve. As well, critics of clean energy, especially those from the oil industry, argue vehemently that the sun doesn't shine at night and winds don't blow 24 h a day. They say that the Achilles heel of these technologies is the ability to store energy, because batteries are prohibitively expensive and big and bulky.

The critics are wrong on this front as well, because the cost of energy storage is also plummeting. Since 1990, the cost of batteries has fallen by a factor of roughly twenty. On current trends, the price of batteries and other energy-storage techniques will fall to just a few cents per kWh by the time solar and wind have matured, making energy from the sun and wind available 24/7 and cheaper than electricity from any other source.

The advances are exceeding expectations. In a study published in *Nature Climate Change*, Bjorn Nykvist and Mans Nilsson, of the Stockholm Environment Institute, documented that, from 2007 to 2011 average battery costs for battery-powered electric vehicles fell by about 14% a year (Nykvist and Nilsson, 2015). This decline put battery costs in 2016 right around the level that the International Energy Agency predicted they would reach in 2020. Electric vehicles are fast reaching the point at which they will cost substantially less to operate, from cradle to grave, than gasoline-fueled ones. And the same technology that is used for car batteries can be used for homes and businesses to store solar energy.

According to the U.S. Department of Energy (DOE), the cost of electric vehicle batteries fell from $1,000 per kWh in 2008 to $268 per kWh in 2015, a 73% reduction in 7 years (International Energy Agency, 2016). Wharton's Mack Institute's Program on Vehicle and Mobility Innovation calculated the cost lithium-ion (Li-ion) battery packs had declined 16% annually between 2007 and 2020, dropping to industry-wide average cost of battery packs of US $144 per kWh in 2020.

By the way, many new solar (and battery) technologies are in development. For example, scientists are experimenting with a new material called perovskite, a light-sensitive crystal that has the potential to be more efficient, less expensive, and more versatile than any solar solutions to date. From 2009 to 2017, perovskite's conversion efficiency increased from 3.8 to 22.7%, making it the fastest-developing technology in the history of photovoltaics (Manser et al., 2016). In June 2018, Oxford PV announced that its perovskite-silicon tandem solar cell had achieved a 27.3% conversion efficiency, as certified by the Fraunhofer Institute for Solar Energy Systems (Oxford PV, 2018). Researchers in Japan forecast a maximum potential efficiency exceeding 38% in a chalcogenide perovskite/crystalline Si tandem architecture.

## How this benefits everyone, everywhere

The effect of these advances is not limited to the developed world; it is anywhere where people can put a solar panel on a roof. Free power will trickle down even to remote villages, with profound consequences. This is already happening.

In Africa, 1.2 billion people have no connection to a power grid, and another 2.5 billion can get power only intermittently. To make matters worse, the lack of viable electrical options creates perverse side effects. People use kerosene for lamps, a dirty fuel that, according to the *Economist*, costs $10 per kWh of energy that it provides—significantly more costly than the same unit of power in the West on a modern power grid (Economist, 2015). Worse, kerosene fires are endemic in Africa, and their toxic fumes cause respiratory ailments that kill hundreds of thousands per year.

The plummeting cost of photovoltaic panels, along with the decline in the prices of light-emitting diodes (another semiconductor product), has brought light to more than 20 million Africans in the past decade. The World Bank's Lighting Africa program is doubling sales of approved devices each year (World Bank, 2018). Solar-powered LED lamps with included battery storage sell for $8 (Economist, 2012). That's still a lot of money for the poorest to afford, but it's within reach.

Central power grids will probably never be built to cover all of Africa. Power there will truly be a distributed endeavor. Schools, hospitals, and homes will all be powered by sources on site or nearby. The same happened with landline communications: Africa leapfrogged into cell-phone networks. In some places, these networks are better than those in the United States. By leapfrogging legacy infrastructure and focusing on the future, Africa will be able to take far better advantage of future price declines in solar, LED, and other energy–capture and -saving technologies.

Aside from its effect on lighting, distributed micro-generation in Africa will also allow cheaper charging of cell phones. This is, believe it or not, a major expense for many Africans who lack sources of electrical energy: they pay dearly for electricity at kiosks. By reducing the cost of phone ownership and making voice and data communication cheaper, low-cost electricity boosts a key service that lifts people out of poverty and improves their lives. Information is power: to get the information, you need the power. Within a decade, we should see 50% penetration of solar panels into Africa and total penetration of LEDs or close access to cheap electricity for running small household appliances or charging phones.

So everywhere on Earth, for rich nations and poor nations, there will be light for all, and it will be essentially free. This will lead to many other benefits. And as we have seen from the reverse innovation that Alfredo Zolezzi is doing with PWSS and Airbus, the relatively well off will also benefit from having inexpensive, clean water without plastic residues—as well as nearly free energy for electric cars and homes.

## Free power means a more peaceful planet

Water and energy are the natural resources at the heart of many of the worst global conflicts. In the Ukraine, a core part of the dispute with Russia is over natural-gas pipelines. Japan started World War II in part due to its lack of natural resources, among them oil. India and China are tussling over water rights, a dispute that looks set to radically worsen as China seeks to expand agriculture in its south and India also pushes to grow enough food to satisfy its fast-growing population. China is proposing massive dams on major rivers flowing from China to India and Bangladesh (Ramachandran, 2015).

With cheaper power making water more abundant, even more of the desert may blossom in green edibles. The world has plenty of desert with plenty of natural sunshine for farms. Israel has pioneered desert agriculture, and tomato farms in Arizona are some of the most productive in the world. Adding water to these vast deserts, far cheaper than fertile fields, will allow many arid countries to become efficient producers of crops. Vertical farming also has great potential. Imagine turning those city parking lots that are no longer needed because of self-driving cars into farms that grow organic food with LED lights and artificial–intelligence software—organic because when food is grown in buildings surrounded by glass, we have no use for insecticides or pest control.

In his book *Abundance: The Future Is Better Than You Think*, Peter Diamandis wrote about an era in which all the needs of humanity are met: a world in which no one on Earth suffers from hunger or lacks clean water; a world in which we all have clothes, electricity, cell phones, and housing; and he believes that this is an eminently achievable aim (Manser et al., 2016). I agree with him—if we do things right, if we can find a way of sharing the benefits of technology advances, and if we take the right paths.

Nearly free energy and water will be amongst the biggest boosts to autonomy that humans have enjoyed in history. Energy and water are the key to everything that offers us a more comfortable life. Energy keeps us warm, powers our vehicles, lights our homes, powers our communications systems, and much more. Inexpensive energy will also unlock an endless supply of fresh water and allow us to grow more food.

Combined, energy and water will give us as much as we could ever want or need. In those parts of the world that are poorly governed or have poor infrastructure, inexpensive energy and water will also allow people to experience lives of a quality far closer to that of us in the West and the developed world. There is no autonomy tradeoff; almost free energy and water will give us more autonomy and reduce our dependency. The ease of accessing energy and water will deliver a base level of abundance that will improve the wellbeing of all people on the planet, from the richest to the poorest.

## Author contributions

The author confirms being the sole contributor of this work and has approved it for publication.

## Conflict of interest

The author declares that the research was conducted in the absence of any commercial or financial relationships that could be construed as a potential conflict of interest.

## Publisher's note

All claims expressed in this article are solely those of the authors and do not necessarily represent those of their affiliated organizations, or those of the publisher, the editors and the reviewers. Any product that may be evaluated in this article, or claim that may be made by its manufacturer, is not guaranteed or endorsed by the publisher.
